# Engineering glowing plants: recent progress and future directions for application-oriented design

**DOI:** 10.3389/fbioe.2026.1819614

**Published:** 2026-04-29

**Authors:** Guimeng Cui

**Affiliations:** Department of Interdisciplinary Program in Landscape Architecture/Graduate School of Environmental Studies, Seoul National University, Seoul, Republic of Korea

**Keywords:** bioluminescence, glowing plants, landscape, plant biotechnology, synthetic biology

## Abstract

Bioluminescence is widespread in fungi and animals but naturally absent from higher plants. Early attempts to generate glowing plants relied on substrate-dependent reporter systems, which limited their relevance to laboratory imaging. Recent advances in synthetic biology and metabolic engineering, particularly reconstruction of the fungal bioluminescence pathway, have enabled the development of autonomously glowing plants without external substrates. Recent studies report notable improvements in brightness, controllability, and regulatory flexibility, as well as the emergence of non-transgenic nanomaterial-based luminescence strategies. Compared with systems developed before, which were mainly restricted to low-intensity signals and proof-of-concept demonstrations, current approaches expand application potential toward ornamental horticulture, educational and artistic installations, and exploratory environmental sensing. Despite these advances, several challenges remain, including metabolic burden, variable stability across developmental and environmental conditions, and ecological and regulatory constraints. Future research should focus on rational optimization strategies that balance light output with plant physiological integrity, implement inducible or tissue-specific expression systems, and integrate ecological risk assessment into technological design. This review summarizes recent progress in luminescent plant engineering, highlights differences between early and current systems in application readiness, and outlines key challenges and future directions for applied plant biotechnology.

## 1 Introduction

Bioluminescence, while widespread among marine organisms and fungi, is naturally absent from the evolutionary lineage of terrestrial higher plants. Despite the diversity of plant specialized metabolism, no wild plant species has been identified to possess autonomous light-emitting capabilities. Early attempts to introduce light emission into plants relied on heterologous expression of firefly luciferase as a molecular reporter system, requiring exogenous application of luciferin and producing only faint, experimentally detectable signals (Ow et al., 1986). For decades, plant luminescence remained largely confined to laboratory imaging and gene expression studies, rather than serving as a visible or functional trait.

Recent advances in synthetic biology and metabolic engineering have fundamentally changed this landscape. Unlike traditional bioluminescence systems (e.g., from fireflies) that remain non-autonomous in plants due to the absence of endogenous luciferins (Ow et al., 1986), the elucidation of complete luciferin biosynthesis pathways, particularly the fungal bioluminescence pathway derived from *Neonothopanus nambi*, has enabled the generation of truly autonomously luminescent plants that no longer require external substrates (Kotlobay et al., 2018; Mitiouchkina et al., 2020; Khakhar et al., 2020). The key to this autonomy lies in the fungal bioluminescence pathway’s utilization of caffeic acid, a common intermediate in the plant phenylpropanoid metabolism, allowing the plant to internally synthesize and recycle its own substrates. Consequently, this pathway can be efficiently integrated into plant cells with relatively limited metabolic rewiring. Subsequent optimization through pathway engineering and directed evolution has led to substantial increases in light output and stability, allowing luminescence to become visible to the naked eye under low ambient light conditions (Shakhova et al., 2024; Zheng et al., 2023). These breakthroughs mark a transition from proof-of-concept reporter plants to a new class of engineered organisms capable of sustained, autonomous light emission.

In parallel with genetic engineering strategies, non-transgenic approaches based on nanomaterials and photonic technologies have also emerged. Delivery of luciferase systems using nanoparticles or coating plants with photoluminescent materials can generate short-term but relatively bright light emission without altering plant genomes (Kwak et al., 2017; 2019; Liu et al., 2025). Although these methods lack long-term stability and heritability, they provide complementary routes for studying plant–light interactions and for potential applications in contexts where genetic modification faces regulatory or societal constraints.

Beyond their technological novelty, autonomously luminescent plants raise important questions for plant science regarding metabolic burden, trait stability, and physiological integration. Light production is directly coupled to cellular energy and carbon fluxes, and sustained luminescence may influence growth, stress responses, and reproductive fitness. Understanding how engineered bioluminescence interacts with endogenous plant metabolism therefore represents both a challenge and an opportunity for advancing synthetic biology in multicellular photosynthetic systems.

At the same time, the development of glowing plants has stimulated increasing interest in their potential applications beyond laboratory imaging (Reuter et al., 2020; Li et al., 2021; Piyush et al., 2022; Yu et al., 2025). Proposed uses include ornamental horticulture, indoor and outdoor landscape design, environmental sensing, and educational or artistic installations. Compared with conventional artificial lighting, bioluminescent plants emit cold light without electrical input and can be integrated into living plant structures, offering a unique combination of aesthetic value and biological function. Importantly, current luminescence levels remain far below those required for functional illumination, and glowing plants should be viewed as complementary visual or symbolic elements rather than replacements for existing lighting technologies. Their greatest near-term relevance lies in low-intensity decorative and experimental contexts, where biological light emission itself becomes a novel trait of interest.

This review summarizes recent advances in the engineering of autonomously luminescent plants, including both genetically encoded and non-genetic strategies. I focus on key technological milestones, compare different bioluminescence systems, and discuss their potential applications in ornamental and environmental settings. Finally, I highlight the major biological, technical, and regulatory challenges that must be addressed before glowing plants can move from experimental systems toward broader use in plant biotechnology and applied horticulture.

## 2 Genetically integration of luciferase-luciferin systems for glowing plants

The engineering of glowing plants has progressed from substrate-dependent reporter systems to fully autonomous bioluminescent organisms through successive advances in synthetic biology and metabolic pathway reconstruction. Early efforts introduced the firefly (*Photinus pyralis*) luciferase gene into tobacco and other plant species, enabling a yellowish-green luminescence (550–570 nm) only after exogenous application of luciferin (Ow et al., 1986; Millar et al., 1992). These systems proved valuable as molecular reporters but produced light intensities detectable only under laboratory conditions and were unsuitable for visible or sustained luminescence in whole plants.

A major conceptual advance was achieved with the introduction of complete bacterial bioluminescence operons *luxCDABE*, which encodes both luciferase and the enzymes for luciferin biosynthesis. Transgenic expression of this pathway in plant enabled continuous cyan light emission (∼490 nm) without external substrates (Krichevsky et al., 2010; Cui et al., 2014). However, the bacterial system imposed substantial metabolic burden and exhibited cytotoxicity associated with aldehyde intermediates (Kotlobay et al., 2018), resulting in weak and unstable luminescence and limiting its practical utility in higher plants.

The field was transformed by the elucidation of the fungal bioluminescence pathway from *N. nambi*, which revealed a luciferin biosynthetic route directly linked to the plant phenylpropanoid metabolism via shared intermediates such as caffeic acid (Kotlobay et al., 2018; Stevani et al., 2024; Ge et al., 2025). This metabolic compatibility enabled more efficient integration of bioluminescence into plant cells with reduced physiological disruption. In 2020, independent studies successfully reconstructed this pathway, the core enzymes including Luz, HispS, H3H, and CPH, in multiple plant species, including tobacco, potato, and ornamental plants, producing autonomously green luminescent (∼520 nm) individuals visible to the naked eye without chemical supplementation (Mitiouchkina et al., 2020; Khakhar et al., 2020). These results established fungal bioluminescence as the first broadly applicable system for generating self-sustained luminescent plants.

Subsequent work has focused on enhancing light emiting and stability through pathway optimization and protein engineering. A significant advance came from the development of a hybrid luminescence pathway that combines compact plant-derived hispidin synthases with fungal bioluminescence enzymes. In contrast to the canonical fungal bioluminescence pathway, which requires the massive HispS and its obligatory activator npgA for precursor biosynthesis, this hybrid architecture leverages the plant’s endogenous phenylpropanoid metabolism. By substituting the hispidin biosynthesis pathway with a compact plant type III polyketide synthase (specifically a styrylpyrone synthase such as PpASCL or PzPKS2), the researchers successfully bypassed the requirement for post-translational modification while utilizing host-derived coenzyme A. This drastic reduction in total transgene size not only minimizes the metabolic drain on the host but also enables, for the first time, the delivery of the entire bioluminescent cassette via viral vectors with restricted cargo capacities, such as TMV (Palkina et al., 2024). The optimization of the fungal bioluminescence pathway led to the development of improved variants, notably FBP2 and FBP3, through the directed evolution and rational design of key components. A primary advancement involved the engineering of the Luz luciferase that exhibited enhanced thermal stability and catalytic efficiency, and the engineering of the H3H enhanced the brightness of the pathway (Shakhova et al., 2024). Complementary metabolic engineering strategies further amplified photon emission by adjusting precursor flux and reducing competing metabolic sinks. Specifically, by incorporating a high-efficiency BnC3′H1 enzyme from *Brassica napus* and NPGA which produces more caffeic acid, the researchers achieved enhanced auto-luminescent plants that sufficient to illuminate its surroundings and visualize words clearly in the dark (Zheng et al., 2023). Overall, these findings provide a robust tool for real-time metabolic monitoring and the development of high-brightness ornamental glowing plants.

## 3 Engineered glowing plants with fluorescent proteins

While autonomous bioluminescence offers substrate-free light emission, it is fundamentally distinct from conventional optical tools such as fluorescent proteins and synthetic probes. Aside from bioluminescence, fluorescent proteins and synthetic fluorescent probes are the most prevalent optical tools in plant science. Previous transgenic plants expressing fluorescent proteins or nano-lantern reporters emit light only under external excitation and therefore do not constitute true bioluminescent organisms (Sasaki et al., 2014; Chin et al., 2018; Furuhata et al., 2020). A recent study, however, demonstrated an engineered nano-lantern capable of autonomous, substrate-free light emission in multiple colors in plant hosts, enabling multiplexed imaging without external excitation (Kusuma et al., 2024; Hattori et al., 2025). Such multicolor capability offers a promising avenue for future development of visually diverse luminescent plants and for advanced *in vivo* imaging applications.

Aside from bioluminescence, fluorescent proteins and synthetic fluorescent probes are the most prevalent optical tools in plant science. However, these systems are fundamentally different from autonomous bioluminescence as they require external excitation light (e.g., UV or blue light). For fluorescent proteins, a major disadvantage in glowing plant applications is phototoxicity (Icha et al., 2017). High-intensity excitation light triggers the production of reactive oxygen species, leading to cellular damage and impaired plant physiology (Laissue et al., 2017).

Collectively, genetically engineered approaches illustrate a trajectory from reporter-based luminescence toward metabolically integrated, self-sustaining light emission in plants. The fungal bioluminescence pathway currently offers the most promising balance between brightness, metabolic compatibility, and genetic stability. Nevertheless, substantial challenges remain, including achieving sufficient light intensity in woody or perennial species, minimizing fitness costs associated with continuous photon production, and maintaining stable expression across developmental stages and environmental conditions. Addressing these issues will be essential for translating bioluminescent traits from model species to horticulturally and ecologically relevant plants.

## 4 Nanomaterial and photonic approaches for glowing plants

In addition to genetic engineering, non-transgenic strategies have been developed to induce light emission in plants through the external introduction of luminescent systems or photonic materials. These approaches provide alternative routes for generating visible plant luminescence without permanent modification of plant genomes and are therefore of interest in contexts where regulatory, ecological, or public acceptance constraints limit the use of genetically engineered organisms.

One representative example is the so-called “nanobionic” light-emitting plant, in which luciferase enzymes, luciferin substrates, and cofactors are delivered into intact plant tissues using engineered nanoparticles (Kwak et al., 2017; 2019). By encapsulating these components in silica, polymer, or chitosan-based carriers, transient but relatively bright luminescence can be achieved in whole leaves for periods ranging from hours to days. This strategy demonstrates that plant tissues can serve as bioreactors for light production without genetic transformation, but its dependence on repeated substrate loading and limited duration restricts its use to short-term or experimental applications.

Another emerging method relies on photoluminescent or afterglow materials, such as strontium aluminate–based phosphors, that can be introduced into or onto plant tissues. These materials absorb ambient light during the day and re-emit it gradually in the dark, producing visible afterglow in treated plants (Liu et al., 2025). Compared with enzymatic nanobionic systems, photonic coatings offer greater flexibility in emission color and do not require metabolic energy from the plant. However, their luminescence is inherently transient and depends on prior light exposure, and the long-term effects of repeated material application on plant physiology and surrounding ecosystems remain largely unexplored.

Nanomaterial and photonic approaches thus differ fundamentally from genetically encoded bioluminescence. Whereas transgenic strategies aim to establish heritable, metabolism-coupled light production, non-genetic methods generate externally driven luminescence that is temporary and user-controlled. Importantly, nanomaterial-based approaches excel in spectral versatility, as they can be engineered to emit light across the full visible spectrum, providing a broader color palette than current biologically restricted pathways. These systems can produce higher short-term brightness and allow rapid prototyping of glowing plants for demonstration or artistic purposes, but they lack the stability, scalability, and biological integration required for long-term deployment.

However, challenges arise from metabolic dilution and phytotoxicity (Besten et al., 2026). Most probes are optimized for animal cells and may exhibit poor cell wall penetrability or cytosolic instability in plants. Continuous imaging is often hampered by the need for repeated exogenous application, which is impractical for sustainable, large-scale ornamental or sensing applications. In contrast, autonomous systems like the fungal bioluminescence pathway platform operate without external intervention, offering a “zero-background” signal that is not obscured by the plant’s natural autofluorescence (Donaldson, 2020).

From a plant science perspective, these non-transgenic techniques provide valuable experimental platforms for studying light perception, stress responses, and plant–material interactions under luminescent conditions. At the same time, they highlight key trade-offs between controllability and sustainability. While avoiding genetic modification may ease regulatory barriers, the need for continuous material input and the uncertainty surrounding environmental persistence limit their applicability for large-scale or permanent use. Consequently, nanomaterial-based luminescence should be viewed as complementary to genetically engineered approaches rather than as substitutes for autonomous bioluminescent plants.

## 5 Application potential of luminescent plants

The concept of light-emitting plants has long held appeal beyond basic research, with potential uses ranging from ornamental horticulture to environmental sensing. Early engineered bioluminescent plants were primarily evaluated as proofs of principle: they demonstrated that autonomous light emission in photosynthetic organisms is feasible, but low signal intensity and limited control over expression confined their relevance to controlled laboratory environments. Only with recent advances in pathway optimization, regulatory elements, and alternative luminescence strategies has the prospect of practical application begun to take shape.

To clarify the technological diversity and application readiness of luminescent plant systems, the major engineering strategies can be compared in terms of mechanism, brightness, stability, and practical constraints (Table 1).

**TABLE 1:** Comparison of technological routes and application potential of luminescent plants.

Technological route	Principle	Brightness level	Duration	Autonomous luminescence	Color tuning mechanism	Representative applications	Main limitations
Fungal bioluminescence pathway	Reconstruction of the fungal caffeic acid–based luciferin biosynthetic pathway integrated into plant phenylpropanoid metabolism	Medium–high (visible under low ambient light)	Continuous and heritable	Yes	Fixed by luciferase structure; shifted via BRET	Autonomous glowing plants, real-time biological visualization, physiological monitoring	Insufficient brightness for illumination; metabolic burden on host plants
Nanobionic light-emitting plants	Delivery of luciferase systems or luminescent substrates into plant tissues using engineered nanoparticles	Moderate (visible for several hours)	Transient (hours to days)	No	Size-dependent quantum confinement (quantum dots)	Demonstration lighting, experimental sensing platforms, artistic installations	Short duration; requires repeated material loading; limited scalability
Transgenic reporter-based luminescence (bacterial/firefly systems)	Expression of heterologous luciferase genes requiring exogenous substrates	Low	Substrate-dependent	No/weak	Enzyme engineering; orthologous variants	Gene expression reporters, laboratory imaging	Dependence on external luciferin; low light output
Hybrid and photonic systems	Combination of bioluminescent reactions with photonic or fluorescent materials to enhance or shift emission spectra	Tunable	Programmable	Partial	Module substitution; phosphor doping	Biosensing, stress reporting, experimental monitoring	System complexity; long-term stability not established

### 5.1 Progress in light output and visibility

Most early autonomously glowing plants exhibited light levels detectable only with sensitive cameras or in near-dark conditions, limiting their utility for visible applications outside specialized settings. The bacterial lux system and early fungal pathways produced luminescence orders of magnitude below ambient lighting thresholds and imposed significant metabolic burdens on host plants.

In contrast, recent studies have demonstrated substantial improvements in light output through both genetic and non-genetic strategies. Enhanced fungal bioluminescence pathways (e.g., optimized constructs FBP2/FBP3) have achieved sustained luminescence that is visible under low ambient light without chemical substrates, owing to improved enzyme kinetics and expression cassettes tailored to plant cellular contexts (Shakhova et al., 2024). These developments mark a qualitative increase in application potential, enabling visually appreciable light emission in ornamentals and model species without external excitation (Palkina et al., 2024).

Non-genetic nanomaterial and photonic approaches have also advanced. While still transient, optimized delivery systems and improved phosphorescent materials produce comparatively higher short-term brightness, facilitating demonstration projects, artistic installations, and experimental studies of light responses in plants. These strategies, when combined with genetic systems, offer a toolkit for tuning brightness across a broader dynamic range than was previously achievable.

### 5.2 Controlled expression and on/off switching

A major limitation of early bioluminescent plants was the lack of temporal control, as luminescence was constitutively coupled to host metabolic activity. To date, most autonomously luminescent systems rely on continuous expression of pathway enzymes, and robust on/off switching of visible light emission has not yet been widely achieved in whole plants.

Recent studies in plant synthetic biology have nevertheless demonstrated the feasibility of regulating multigene pathways using inducible or developmentally controlled promoters, providing a conceptual framework for future control of luminescence traits. For example, chemically inducible or tissue-specific regulatory elements have been successfully applied to complex metabolic pathways and reporter systems, suggesting that similar strategies could be adapted to bioluminescent pathways (Belcher et al., 2020; Yasmeen et al., 2023).

Rather than enabling precise real-time switching, current control strategies mainly allow partial modulation of luminescence intensity or spatial restriction of expression to specific tissues or developmental stages. Such regulation is more realistic for near-term applications, including reducing metabolic burden, improving trait stability, and tailoring luminescence to ornamental or experimental contexts. In this sense, controlled expression should be viewed as an emerging design principle rather than a fully mature technology for dynamic light switching in plants.

### 5.3 Species-specific constraints and transformation efficiency

A significant bottleneck in the translational use of bioluminescent traits is the variability in transformation efficiency and metabolic compatibility across different plant taxa. While the fungal bioluminescence pathway has been successfully demonstrated in model species (Mitiouchkina et al., 2020; Khakhar et al., 2020), extending these traits to horticulturally significant monocots or slow-growing woody perennials remains technically demanding. These non-model species often exhibit lower expression levels of the multi-gene bioluminescence cascade and may require intensive codon optimization and the use of specialized strong or tissue-specific promoters to achieve visible light output. In contrast, nanomaterial-based strategies offer a quick-access route to luminescence in diverse species, as they bypass the need for genomic integration, though they lack the heritability required for permanent ornamental varieties.

### 5.4 Non-transgenic strategies and regulatory advantages

Applications that require rapid deployment or must comply with strict regulatory environments benefit from non-genetically modified (non-GMO) approaches. Nanobionic and photonic methods introduced in recent years (e.g., engineered nanoparticles and phosphor coatings) can confer luminescent properties to plants without altering their genomes. These techniques present clear policy and public acceptance advantages in regions where GMO cultivation is restricted or controversial, enabling short-term demonstrations and pilot projects that would be infeasible with transgenic plants.

Although non-genetic luminescence is inherently transient and dependent on repeated material application, its independence from stable genomic modification circumvents many regulatory hurdles and reduces concerns related to gene flow and long-term ecological impacts. This positions non-GMO luminescent plants as attractive candidates for decorative horticulture, science outreach, and controlled environment agriculture where regulatory constraints would otherwise limit adoption.

### 5.5 Emerging use cases and contexts

Taken together, these advances support a spectrum of potential applications that extends well beyond early conceptual discussions. (1) Ornamental horticulture: Bioluminescent traits can differentiate ornamental varieties and create novel aesthetic effects for indoor and outdoor gardens (Li et al., 2021). (2) Educational and artistic installations: Dynamic or inducible luminescence offers engaging platforms for conveying biological concepts to diverse audiences. (3) Environmental sensing and reporting: Conditional luminescence tied to physiological or environmental triggers may serve as a visual readout for stress responses or nutrient status in experimental settings (Volkov and Ranatunga, 2006). (4) Complementary landscape elements: In low-light or twilight conditions, luminescent plants may function as decorative accents rather than primary light sources, enriching plant collections without significant energy input (Li et al., 2021).

While true functional illumination at levels comparable to engineered lighting remains a distant goal, the cumulative progress of the past 2 years significantly expands the range of contexts in which luminescent plants can be meaningfully deployed.

## 6 Key challenges and future optimization

Despite recent progress in engineering luminescent plants with improved brightness and controllability, several fundamental challenges continue to limit their broader application. These challenges span biological constraints, technical scalability, ecological considerations, and regulatory feasibility. Addressing them will require integrated optimization strategies that combine synthetic biology, plant physiology, and materials science.

### 6.1 Metabolic burden and physiological integration

A central challenge in autonomously luminescent plants is the metabolic cost associated with continuous photon production. Bioluminescence depends on precursor availability, enzyme activity, and cellular energy flux, which compete with endogenous growth and stress-response pathways (Reuter et al., 2020; Li et al., 2021). Although recent fungal pathway–based systems exhibit improved metabolic compatibility with plant phenylpropanoid metabolism, sustained luminescence may still impose fitness penalties, particularly in long-lived or woody species (Wurtzel and Kutchan, 2016).

To produce the same level of brightness as traditional streetlights, glowing plants must emit over 1,000 lumens through a firefly-based bioluminescence system. According to theoretical estimation for the firefly luciferase-luciferin system, a fast-growing tree would need to allocate only about 0.3% of its stored energy to generate sufficiently bright bioluminescence at night (Reeve et al., 2014). While these calculations highlight the metabolic feasibility of high-intensity bioluminescence, it should be noted that they are conceptual and based on the firefly system. Besides, using the fungal bioluminescence pathway in plants may involve different metabolic costs.

Future optimization should focus on balancing light output with host physiology. Strategies may include fine-tuning promoter strength, restricting luminescence to specific tissues or developmental stages, and employing inducible or circadian-regulated expression systems. Such approaches would reduce unnecessary metabolic load while preserving functional or aesthetic utility. Quantitative assessment of growth, reproduction, and stress tolerance in luminescent plants will be essential to establish safe operational thresholds for long-term expression.

### 6.2 Brightness enhancement and color alterations

Although recent advances have achieved visible luminescence under low ambient light, current emission levels remain far below those required for functional illumination. Improvements in brightness have largely relied on enzyme engineering and pathway optimization, but further gains may depend on enhancing photon extraction and optical efficiency rather than metabolic flux alone (Kusuma et al., 2025).

Potential directions include targeting luminescence to surface tissues, modifying cellular microenvironments to reduce photon absorption or scattering, and coupling bioluminescent reactions with fluorescent or photonic elements that shift emission spectra toward higher visual sensitivity via synthetic biology (Purnick and Weiss, 2009). Importantly, brightness enhancement must be pursued cautiously to avoid exacerbating metabolic stress or compromising plant viability.

Beyond brightness, spectral tuning represents a significant frontier in plant bioluminescence. While the fungal pathway natively emits green light, expanding the color palette is essential for aesthetic diversity and multiplexed sensing (Mujawar et al., 2024). Recent strategies include coupling bioluminescent reactions with fluorescent proteins through bioluminescence resonance energy transfer (BRET) to shift emission toward yellow or red wavelengths (Tsuboia and Jin, 2019). However, achieving efficient, autonomous multicolor emission in whole plants remains a technical bottleneck due to the complexity of multi-gene coordination and potential interference with plant pigment absorption.

### 6.3 Stability across development and environments

Trait stability remains a major bottleneck for translational use. Luminescence intensity often varies with developmental stage, circadian rhythms, and environmental conditions such as temperature, nutrient availability, and water status. In perennial or deciduous plants, seasonal dormancy and tissue senescence further constrain year-round performance.

Future work should emphasize genetic designs that confer robust and predictable expression profiles across time and environments. Genome integration strategies that minimize transgene silencing, along with modular pathway architectures that allow adaptive tuning, may improve consistency. Long-term field or semi-field studies will be critical to evaluate how engineered luminescence behaves outside controlled laboratory conditions.

### 6.4 Ecological and biosafety considerations

Even low-level nocturnal light can influence insect behavior, pollination dynamics, and plant–animal interactions (Gaston et al., 2013; Kyba et al., 2017; Sanders et al., 2021). Large-scale deployment of luminescent plants could therefore introduce ecological perturbations that are not yet fully understood. Moreover, gene flow from transgenic ornamentals to wild relatives remains a potential concern in certain ecosystems.

Future optimization should incorporate ecological risk assessment alongside technical development. Spatial confinement strategies, sterility designs, and the selection of non-invasive host species may mitigate unintended impacts. Parallel exploration of non-genetic luminescence methods offers an alternative route for minimizing long-term ecological uncertainty, particularly in sensitive environments.

### 6.5 Regulatory and translational pathways

Regulatory barriers continue to shape the feasibility of real-world applications (Gleim and Smyth, 2018). While recent commercialization of luminescent ornamentals in limited markets demonstrates progress, genetically engineered plants remain subject to strict biosafety frameworks in many regions. In this context, non-transgenic nanomaterial and photonic approaches present practical advantages by avoiding genomic modification and facilitating short-term or reversible implementations.

Future optimization should therefore proceed along dual tracks: advancing genetically encoded systems for stable, heritable traits while simultaneously refining non-GMO strategies for rapid deployment and demonstration purposes. Hybrid approaches that integrate controllable luminescence modules without permanent genetic alteration may further expand the range of acceptable applications (Schaumberg et al., 2016).

Moving forward, the development of luminescent plants will benefit from a shift from proof-of-concept demonstrations to application-oriented design principles. This includes defining target brightness ranges, acceptable metabolic costs, and context-specific performance criteria for ornamental, experimental, or sensing applications. Advances in computational pathway modeling, synthetic regulatory circuits, and high-throughput phenotyping will support rational optimization rather than empirical trial-and-error.

Ultimately, the success of luminescent plants will depend not only on achieving higher light output, but also on ensuring biological compatibility, environmental safety, and regulatory viability. Continued interdisciplinary efforts are needed to translate recent technical breakthroughs into robust plant traits suitable for horticulture and applied plant biotechnology.

## 7 Discussion

The past decade has witnessed a transition in luminescent plant research from substrate-dependent reporter systems to autonomously glowing organisms enabled by advances in synthetic biology and metabolic engineering. In particular, the elucidation and optimization of the fungal bioluminescence pathway have established a biologically compatible framework for integrating light production into plant metabolism. Recent studies further demonstrate substantial progress in brightness enhancement, controllable expression, and non-genetic luminescence strategies, marking an important step toward application-oriented designs rather than purely experimental proofs of concept.

Compared with earlier generations of bioluminescent plants reported earlier, which were largely restricted to laboratory imaging and demonstration purposes, current systems exhibit improved visibility, regulatory flexibility, and broader contextual relevance. These advances expand the feasible application space to include ornamental horticulture, educational and artistic installations, and experimental environmental sensing. At the same time, luminescent plants should be viewed as complementary biological traits rather than substitutes for conventional lighting technologies, given that their emission levels remain far below those required for functional illumination.

Looking forward, future research should prioritize rational optimization strategies that balance light output with plant physiological integrity. Key directions include tissue-specific or inducible expression systems to reduce metabolic burden, further enhancement of photon efficiency through enzyme and pathway engineering, and long-term evaluation of trait stability under variable environmental conditions. In parallel, non-transgenic nanomaterial and photonic approaches provide valuable alternative routes for short-term deployment and regulatory compliance, particularly in regions where genetically modified plants face significant barriers.

Equally important will be the integration of ecological risk assessment and regulatory considerations into technological development. The deployment of luminescent plants in real-world settings requires careful evaluation of their potential effects on plant–animal interactions and surrounding ecosystems, as well as transparent communication of their intended roles and limitations. Advances in containment strategies, such as sterility designs or spatial confinement, may further support safe and responsible implementation.

In summary, luminescent plants represent a unique convergence of plant biology, synthetic biology, and applied biotechnology. The recent acceleration in technical capability suggests that their future impact will depend less on achieving maximal brightness than on achieving biological compatibility, controllability, and societal acceptability. Continued interdisciplinary efforts will be essential to transform glowing plants from scientific curiosities into robust traits for ornamental, experimental, and educational applications.
